# Effects of individuals’ esthetic expectations and the classifications of dentofacial deformities on patients’ depression: a cross-sectional study

**DOI:** 10.3389/fpsyt.2025.1505961

**Published:** 2025-03-20

**Authors:** Jingjun Wang, Yanglu Tang, Mingjun Ren, Wenli Zhao, Yuanyan Bai

**Affiliations:** State Key Laboratory of Oral Diseases & National Center for Stomatology & National Clinical Research Center for Oral Diseases & Dept. of Oral and Maxillofacial Surgery, West China Hospital of Stomatology, Sichuan University, Chengdu, China

**Keywords:** depression, esthetic expectations, dentofacial deformities, mental health, psychological states

## Abstract

**Background:**

Dentofacial deformity (DFD) is a disease in which the maxillary complex markedly diverges from normal proportions. The incidence of DFD is approximately 20% worldwide, and patients with DFDs are at increased risk for depression. Attention should be given to depression in patients with DFDs. However, factors affecting depression in patients with DFDs remain unclear. Previous studies have suggested that the type of DFD and esthetic expectations may influence patient depression, but few studies have clarified the effects of the type of DFD and esthetic expectations on patient depression.

**Methods:**

A total of 471 patients with DFDs were enrolled. The diagnosis of DFD was made by two maxillofacial surgeons according to the Angle’s classification. The visual analog assessment scale of esthetic expectations was used to assess patients’ esthetic expectations. The 9-item Patient Health Questionnaire depression module was utilized to explore patient depression. SPSS 26.0 was used to analyze the data in this study.

**Results:**

Compared with DFD patients who had a master’s degree or above, those who had a high school education were more prone to depression (OR=3.848, 95% CI: 1.546-9.574). Compared with Class II DFDs, Class III DFDs were associated with a greater risk of depression (OR=1.458, 95% CI: 1.007–2.078). Compared with those who had extremely low esthetic expectations, those who had extremely high esthetic expectations (OR: 2.25, 95% CI: 1.053–4.086) were more prone to depression.

**Conclusions:**

Patients who had a high school education, were diagnosed with Class III DFDs, and had higher esthetic expectations had a greater risk of depression. The above populations may need more psychological support.

## 1 Introduction

Dentofacial deformity (DFD) describes a condition in which the maxillary and mandibular markedly diverge from normal proportions, which in turn results in an altered relationship between the teeth and the jaws (1). DFDs are a series of disorders (congenital or acquired) that can lead to facial deformities and functional aberrations in the stomatognathic system, further leading to the development of functional problems such as chewing disorders, swallowing dysfunction, and obstructive sleep apnea (2, 3). Such dysfunction can severely affect a patient’s quality of life (1, 4).

In addition to the impact of DFDs on occlusion, metabolism, and other physical functions, the impact of DFDs on patients’ mental health has also attracted the attention of scholars in recent years (5, 6). A considerable number of people with DFDs have reported psychological distress, which may arise from external circumstances, such as ridicule and certain stereotypes, or from a patient’s internal psychological problems (1, 7–9). On the one hand, DFD patients have demonstrated greater anxiety, social maladaptation, and lower esteem than the general population On the other hand, severe psychiatric disorders can lead to metabolic and behavioral abnormalities, which in turn increase the risk of depression in DFD patients (10, 11). Therefore, the psychological status of patients with DFDs should be considered.

Previous studies have shown that patients with DFDs are at increased risk of severe psychiatric disorders such as depression (12). Collins et al. reported that 42% of patients with DFDs experience depression (13). Depression is a risk factor for serious physical illnesses such as heart disease and cancer (14, 15) and is one of the most common causes of disability in patients who undergo facial plastic surgery (16). In addition, orthognathic surgery is the major treatment for DFDs; preoperative depression in patients with DFDs is closely related to surgical outcomes and the extent of improvement in the postoperative psychological state, and a surgeon may not be able to perform surgery successfully when a patient has severe preoperative depression (17, 18). Given the severe impact of depression on individuals’ psychological health and the effectiveness of treatment, greater attention should be given to depression in patients with DFDs.

However, recent research on the link between depression and DFDs is less adequate than that for other oral diseases (e.g., periodontitis) (6, 19–21). Theories on how DFDs can lead to depression in patients remain controversial (13, 22, 23). For example, Collins et al. reported that DFD is a risk factor for depression and anxiety, whereas Basso and Frejman noted that DFDs can lead to neuroticism rather than depression and anxiety (3, 22, 24). Therefore, whether DFDs can lead to depression in patients’ needs to be further explored.

As there are many types of DFDs with different clinical manifestations, the effects of different types of DFDs on patients’ physical and mental health differ (12, 25, 26). For example, patients with Class II DFDs are at a greater risk of temporomandibular joint disorders and airway stenosis than those with Class III DFDs, and these factors are also significantly associated with depression. Conversely, for example, patients with Class III DFDs have a more negative view of themselves than those with Class II DFDs. Combining different classes of DFDs for analysis may yield inconclusive results. Further analysis of different classes of DFDs may contribute to a more comprehensive understanding of their relationships with depression (6). Exploring the impact of various types of DFDs on patients’ depression can help to deepen the understanding of the relationship between DFDs and patients’ depression and provide targeted psychological support for different types of patients to promote patients’ psychological health and surgical outcomes.

Furthermore, DFDs often severely affect a patient’s facial esthetics (27). Improving one’s facial esthetics is one of the primary reasons why patients seek treatment (28). Esthetic expectations are defined as the extent to which one believes that one’s appearance can be improved by certain treatments (27). Esthetic expectations can be positively interpreted as one’s rational expectations toward a bright future or negatively interpreted as unrealistic expectations (29). Previous studies have indicated that an individual’s psychological status is closely related to their esthetic expectations (27). For example, Möller et al. reported that higher esthetic expectations were associated with mental stress among individuals who underwent plastic surgeries (30). Investigating the relationships between the esthetic expectations of DFD patients and patients’ mental health is instrumental in revealing the factors influencing the mental status of DFD patients. However, to our knowledge, the relationship between esthetic expectations and depression in patients with DFDs remains unclear, and few studies have explored how esthetic expectations in patients with DFDs may affect patient depression.

Therefore, this study aimed to investigate the factors influencing depression among DFD patients and to reveal the effects of different classes of DFDs and esthetic expectations on patient depression to provide targeted psychological interventions based on patients’ disease classes and expectations to reduce the incidence of depression in patients with DFDs.

## 2 Materials and methods

### 2.1 Study design

This was a cross-sectional study.

### 2.2 Samples and settings

This study was conducted from 2024.01 to 2024.05 at West China Stomatology Hospital of Sichuan University. The initial sample size (384) was calculated with the following equation:

$N=Z^{2}\times \frac{\left(P\times \left(1-P\right)\right)}{E^{2}}$ (Z = 1.96, E = 5%, and P = 0.5). Considering the possibility of sample dropout, the overall sample size should be 1.2 times the calculated sample size, i.e., 460 participants. To ensure the representativeness of the sample, the following strict inclusion criteria were formulated: (1) patients aged between 18 and 65 years; (2) patients whose permanent dentition was complete except for third molars; (3) patients diagnosed with Class II or III DFDs by two physicians at a tertiary care hospital; and (4) patients who were informed and consented to participate in this study. The following exclusion criteria were applied: (1) Patients with other types of DFDs (except for sagittal displacement), such as laterognathism of the mandible, hemimandibular hypertrophy, unilateral micrognathia, hemifacial microsomia, and hemifacial atrophy et al; (2) patients with tooth loss (other than the third molars) in the permanent dentition; (3) patients with previous orthodontic treatment; (4) patients with temporomandibular joint disorders, craniofacial deformities such as cleft lip and cleft palate, severe periodontal disease, and/or severe tooth wear or mispositioning; (5) patients suffering from cognitive impairment and/or psychiatric disorders (for example, individuals with schizophrenia or dementia) and those with an inability to communicate due to severe psychiatric disorders; and (6) patients suffering from severe somatic disorders such as cancer, coronary heart disease, and/or aortic dissection.

**Figure 1:**
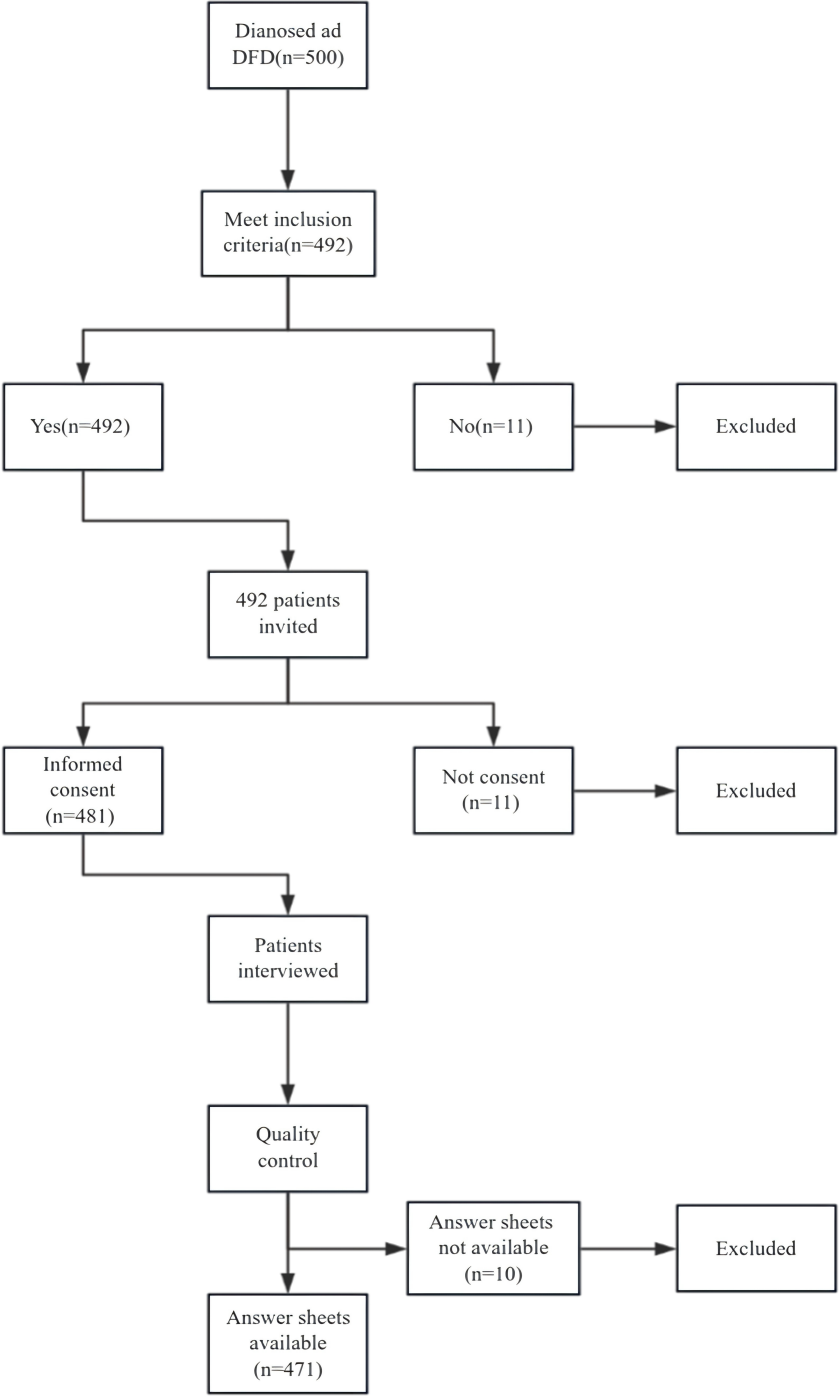
Sampling process.

A total of 500 patients were diagnosed with different types of DFDs at the West China Stomatology Hospital of Sichuan University. A total of 492 patients were invited to participate in this study after meeting the inclusion criteria. A total of 481 patients agreed to participate in this study. Finally, 471 patients with available answer sheets were included in this study. The details of the sampling process are presented in **Figure 1**.

### 2.3 Data collection

An online questionnaire was developed by using WJX software to collect data (), which included three components: patients’ demographic characteristics, patients’ esthetic expectations, and patients’ depression status. Before the formal investigation, the aims, methodology, and precautions of this survey were explained to the participants by one researcher. Then, each participant signed an informed consent form. The online questionnaire was subsequently forwarded to the participants by the researcher for completion.

Strict quality control measures were adopted to ensure the authenticity and rigor of this study. For example, a questionnaire could be submitted only if it took a participant more than two minutes to complete it. The participants could submit the answer sheet only if all the questions were completed. Questionnaires in which only one option for all the items was selected were excluded. Questionnaires in which opposite answers were selected for similar questions were also excluded. All questionnaires were automatically reviewed by the software and then manually verified by the researcher. Overall, 471 questionnaires were collected.

### 2.4 Measurements

#### 2.4.1 Demographic characteristics

A demographic characteristics questionnaire was developed to collect patients’ general information, including sex, age, educational background, monthly income, and marital status.

#### 2.4.2 Classifications of DFDs

According to Angle’s classification system, malocclusions can be categorized as neutral malocclusions, distal–medial malocclusions, and proximal–medial malocclusions using the maxillary first permanent molar as a baseline (31). When the distal and mesial relationships of the maxillary and mandibular jaws and dental arches are out of alignment, the mandibular and mandibular dental arches are in the distal position, and the molars are in a distal relationship, this is called Class II malocclusion. For example, maxillary (vertical/anterior–posterior) excess, mandibular (vertical/left–right/anterior–posterior) deficiency, and maxillary (vertical/anterior–posterior) excess combined with mandibular deficiency are all Class II malocclusions. When the distal and mesial relationships of the maxillary and mandibular jaws and dental arches are out of alignment, the mandibular and mandibular dental arches are in the mesial position, and the molars are in a mesial relationship, this is called Class III malocclusion. For example, mandibular (vertical/anterior−posterior) excess, maxillary (vertical/anterior−posterior) deficiency, and maxillary anterior−posterior deficiency combined with mandibular excess are all Class III malocclusions (31).

X-ray lateral cephalometric radiographs were taken, and the patient was instructed to sit in an upright position with both eyes looking straight ahead. The eye and ear planes were adjusted to be parallel to the ground plane by using a cephalometric positioning frame. Patients were also instructed to close their upper and lower lips, breathe calmly and evenly, and bite their posterior teeth lightly in the orthodontic position. Based on the relative positional relationship between the maxillary and mandibular regions, the dentofacial pattern was classified into three types by measuring the size of the ANB angle using X-ray cephalograms: ANB angles between 0° and 5° were defined as Class I DFDs, ANB angles greater than 5° were defined as Class II DFDs, and ANB angles less than 0° were defined as Class III DFDs.

#### 2.4.3 Aesthetic expectations

This study used a scale developed by Nie et al. to assess the esthetic expectations of patients with DFDs (31). The patients used a visual analog assessment scale to score their current appearance on a scale from 1-10; after completing the first scoring, the patients used a visual analog assessment scale to score their desired appearance after the surgery, and the difference between the two scores was the patients’ esthetic expectation score (31). Patients’ esthetic expectations were categorized based on the patient ratings as extremely low, low, high, or extremely high.

#### 2.4.4 Depression

The Patient Health Questionnaire-9 (PHQ-9) was developed at Columbia University to screen and measure the severity of depression, which is consistent with the Diagnostic and Statistical Manual of Mental Disorders, fourth edition (DSM-4) diagnostic criteria for depression. The scale is a self-assessment scale that consists of nine items scored on a four-point Likert scale. A response of “not at all” was given a score of 0, and a response of “almost every day” was given a score of 4. The sum of the scores of all the items is the total score of the scale. PHQ-9 scale scores of 5–9, 10–14, 15–19, and 20–27 indicate mild, moderate, moderate, severe, and major depression, respectively.

### 2.5 Data analysis

IBM SPSS 26.0 was used to analyze the data in this study. Frequencies and percentages were used to analyze participants’ demographic characteristics, esthetic expectations, and depression status. Differences in depression between groups were detected by the chi-square test, with a significance level of P< 0.05 considered statistically significant in multiple tests. Multivariate logistic regression was used to detect the relationships between the selected factors and patients’ depression status.

### 2.6 Ethical considerations

Before the research was conducted, all the details about this study, including the research objectives, research tools, and participant information, were carefully reviewed by the Ethical Committee of West China Stomatology Hospital, Sichuan University. This study was approved by the Ethical Committee of West China Stomatology Hospital, Sichuan University (NO. WCHSIRB-D-2023-333). Furthermore, owing to the sensitivity of the research topic, the privacy of the respondents was strictly protected before the investigation was conducted. Primary data were available only to the researchers. All the data were adopted only for academic research. Before the survey started, all the participants were informed about the research objectives, methods, tools, and any other details and consented to participate in this survey.

## 3 Results

### 3.1 Demographic information

A total of 471 participants were enrolled in this study. Most of the participants were women (337, 71.5%). A total of 30.8% of the participants were between 21 and 25 years old. Nearly half of the participants had obtained a bachelor’s degree (211, 44.8%). A total of 35.9% of the participants had a salary of 5001–10000 yuan per month. Most of the participants (76.9%) were unmarried. All the details about the participants’ demographic characteristics are shown in (**Table 1**).

**Table 1:** Basic information of participants.

Variables	Categories	Depression		χ^2^ (P_a_)
		No N (%)	Yes N (%)	
**Gender**	Men	91 (28.3)	43 (28.9)	0.18 (0.894)
	Women	231 (71.7)	106 (71.1)	
**Age**	<20	79 (24.6)	37 (25.2)	0.86 (0.836)
	21-25	101 (31.5)	44 (29.9)	
	25-30	71 (22.1)	29 (19.7)	
	>30	70 (21.8)	37 (25.2)	
**Education background**	Junior high school and below	22 (6.8)	6 (4.0)	25.58 (P<0.001*)
	High school education	31 (9.6)	37 (24.8)	
	College degree	75 (23.3)	43 (28.9)	
	Bachelor degree	157 (48.8)	54 (36.2)	
	Master degree and above	37 (11.5)	9 (6.0)	
**Monthly income (Yuan)**	≤2000	20 (6.2)	12 (8.1)	7.65 (0.049*)
	2001-5000	90 (28.0)	44 (29.5)	
	5001-10000	107 (33.2)	62 (41.6)	
	10001-15000	51 (15.8)	17 (11.4)	
	≥15000	54 (16.8)	14 (9.4)	
**Marriage status**	Married	248 (58.2)	110 (57.3)	0.69 (0.708)
	Unmarried	69 (16.2)	37 (19.2)	
	Divorced or other	109 (25.6)	45 (23.4)	
**Profiles of DFD**	Class II	211(65.5)	97(56.4)	4.31(0.049*)
	Class III	111(34.5)	52(43.6)	
**Esthetic expectations**	Extremely low	40(12.4)	12(8.1)	8.45 (0.045*)
	Low	74(23.0)	30(20.1)	
	High	57(17.7)	18(12.1)	
	Extremely high	151(46.9)	89(59.7)	
Total		322(68.4)	149(31.6)	

The symbol * indicated that P<0.05, and the bold values indicated the names of variables.

### 3.2 Classification of DFDs

In this study, 308 patients were diagnosed with Class II DFDs, whereas 163 patients were diagnosed with Class III DFDs (**Table 1**).

### 3.3 Esthetic expectations

The results of this study indicated that nearly half of the patients’ esthetic expectations (240, 51.5%) were extremely high, whereas 75 patients’ esthetic expectations were extremely low. All the details about the participants’ esthetic expectations are shown in (**Table 1**).

### 3.4 Depression status

According to the classification criteria for the PHQ-9, scale scores of 0-4, 10-14, 15-19, and 20-27 indicate mild, moderate, moderate-severe, and major depression, respectively. In this study, 322 (68.4%) DFD patients did not have depression, whereas 149 (31.6%) patients had mild depression or above (**Table 1**).

### 3.5 Comparisons of depression among DFD patients by demographic information, DFD class, and esthetic expectations

The results of the chi-square test indicated that demographic information, educational background, and monthly income were related to depression in patients with DFDs. Furthermore, patient depression might be affected by patients’ esthetic expectations and DFD classification (**Table 1**).

The results of logistic regression demonstrated that, compared with DFD patients who had a master’s degree or above, those with a high school education were more prone to depression (OR=3.848, 95% CI: 1.546-9.574). Compared with Class II DFDs, Class III DFDs were associated with a greater risk of depression (OR=1.458, 95% CI: 1.007–2.078). Compared with those who had extremely low esthetic expectations, patients who had extremely high esthetic expectations (OR: 2.25, 95% CI: 1.053–4.086) were more prone to depression (**Table 2**).

**Table 2:** Regression analysis of participants’ depression.

Variables	B	Se	Wals	P	OR	95% CI	
						Lower	Upper
**Educational background (REF: Master degree and above)**			19.623	0.001*			
Junior high school and below	0.034	0.618	0.003	0.957	1.034	0.308	3.47
High school education	1.347	0.465	8.394	0.004*	3.848	1.546	9.574
College degree	0.599	0.437	1.874	0.171	1.82	0.772	4.287
Bachelor degree	0.098	0.416	0.055	0.815	1.103	0.487	2.494
**Monthly income (REF:<2000)**			4.133	0.388			
2001-5000	-0.272	0.436	0.387	0.534	0.762	0.324	1.792
5001-10000	-0.136	0.429	0.1	0.752	0.873	0.377	2.022
10001-15000	-0.504	0.492	1.051	0.305	0.604	0.231	1.583
≥15000	-0.76	0.505	2.265	0.132	0.468	0.174	1.258
**Esthetic expectations (REF: Extremely low)**			6.303	0.098			
Low	0.535	0.416	1.651	0.199	1.707	0.755	3.858
High	0.267	0.454	0.347	0.556	1.307	0.537	3.179
Extremely high	0.811	0.387	4.385	0.036*	2.25	1.053	4.806
**Profiles of DFD (REF: Class II DFD)** Class III DFD	0.506	0.217	1.982	0.047*	1.458	1.007	2.078
**Constant**	-1.605	0.62	6.703	0.01	0.201		

The symbol * indicated that P<0.05, and the bold values indicated the names of variables.

## 4 Discussion

This study investigated the status quo of depression in patients with DFDs, explored the potential factors influencing depression in DFD patients, and revealed the potential effects of the classifications of DFDs, esthetic expectations, and demographic information on patient depression, which is helpful for providing a new vision for psychological interventions in patients with DFDs.

### 4.1 Status quo of depression in patients with DFD

Depression, which is characterized mainly by a low mood, loss of interest, and lack of energy, can not only significantly exacerbate the risk of self-inflicted harm and suicide but also significantly reduce the success rate of orthognathic surgery and exacerbate postoperative pain in patients with DFDs (6, 14, 15, 32). The results of this study revealed that the incidence of depression in DFD patients was 31.6%, which is significantly greater than that reported in the general population and is consistent with the results of Sebastiani et al (6, 9). Since DFDs can increase the risk of depression in patients and the prevalence of DFDs is as high as 20% in the global population, the significance of interventions for existing as well as future depression in the DFD population is highlighted by this study (1, 6, 12).

### 4.2 Demographic information and patient depression

Regression analysis indicated that, compared with patients who had a master’s degree or above, patients who had a high school education were more vulnerable to depression. Similar findings have been obtained in other studies of non-DFD populations (33–35). On the one hand, a higher education level is often associated with better socioeconomic status. According to health inequality theory, people with higher education levels have more psychological and material capital to withstand life’s stresses and are more resilient to risky events, thus reducing their risk of depression (34). On the other hand, there may be differences in the cognition of people with different levels of education. When personal appearance is overly mythologized, highly educated people may be more rational, developing a more objective view of their facial features (36). Therefore, psychosocial interventions should be provided to patients with DFDs according to their cognitive level, and psychological resilience should be promoted in patients with low education and socioeconomic levels.

### 4.3 Classifications of DFDs and patient depression

Regression analysis indicated that, compared with Class II DFD patients, Class III DFD patients were more vulnerable to depression. These results are consistent with those of Burden et al., who reported that patients with Class III DFDs were significantly less confident in their appearance than patients with Class II DFDs (37). Sen et al. reported that, compared with Class II DFD patients, Class III DFD patients held more negative opinions about their profiles and considered themselves even more unattractive (38).

Skeletal Class III malocclusion is one of the most prevalent DFDs and is defined as maxillary retrusion, mandibular protrusion, or a combination of both, and the positions of the maxillary and mandibular jaws in patients with Class III DFDs are opposite those of the normal position (2, 39). In general, Class III DFD patients present with anomalies and severe mental distress due to mandibular protrusion and maxillary deficiency (39). Owing to the location of the jawbone defect, patients with Class III DFDs might have greater degrees of social impairment and physical pain than those with Class II DFDs (38). A greater need for orthognathic surgery and greater improvement in quality of life in patients with Class III DFDs after orthognathic surgery than in those with Class II DFDs have also been reported in previous studies (39–41). Thus, Class III DFD patients may have a greater degree of distress to their appearance, occlusion, perceived low appraisal by others, etc., resulting in greater susceptibility to psychological problems such as depression. To support this hypothesis, the present study further explored the relationship between the type of DFDs and patients’ esthetic expectations, and the results revealed that, compared with patients with Class II DFDs, patients with Class III DFDs had significantly greater esthetic expectations. This explains another aspect of the emergence of the phenomenon.

Therefore, for patients with DFDs, targeted psychological interventions should be provided according to the type and location of the jaw defect. Attention should be given to boosting self-confidence, correcting negative self-perceptions, and improving depression in patients with Class III DFDs.

### 4.4 Esthetic expectations and depression in DFD patients

Regression analysis indicated that, compared with patients whose esthetic expectations were extremely low, patients whose esthetic expectations were extremely high were more vulnerable to depression. The results of this study are consistent with Pikoos et al. (42). Esthetic expectations are defined as the extent to which a patient wishes to improve his or her appearance through surgery (27). Esthetic expectations contribute to enhancing patients’ compliance with surgery while also increasing their risk of psychological disorders, which is a “double-edged sword” (9). Studies of patients undergoing plastic surgery have demonstrated that these patients experience higher levels of emotional distress about their appearance than the general population does and that improving their appearance is the most important motivation for undergoing surgery. When plastic surgery patients have excessive expectations, their risk of depression is significantly greater (42–44).

Patients are more likely to develop somatoform dysmorphic disorders and negative cognition about their appearance when they are overly concerned about their facial defects and present high levels of esthetic expectations (43). Patients may even fall into the vicious circle of “inappropriate cognition– negative emotions–distorted cognition” (45). According to Beck’s cognitive theory of depression, cognitive components and cognitive processes are susceptibility factors for depression. When an individual develops cognitive biases, as soon as a negative event occurs, negative automated thoughts are generated, followed by depression (46).

Therefore, in psychological interventions for patients with DFDs, patients’ esthetic expectations for surgery should be assessed, and unrealistic expectations and biased perceptions should be corrected to reduce the risk of depression in these patients.

### 4.5 Strengths and limitations

Depression is a serious psychiatric disorder that significantly contributes to the global burden of disease. Previous studies have shown that patients with DFDs are at increased risk of comorbid depression. However, the causes of comorbid depression in patients with DFDs remain unclear. The results of this study suggested that a low education level, class III DFD, and high esthetic expectations were risk factors for depression in patients with DFDs and clarified the potential causes of comorbid depression in some patients with DFDs, which can help health care professionals provide targeted psychological interventions according to patients’ different cognitive levels, types of disorders, and expectations and reduce the prevalence of depression in patients with DFDs.

This study has several limitations. First, a convenience sampling method rather than random sampling was used, which may have resulted in bias, and the sample included only 471 patients. This may lead to the sample being unrepresentative. Subsequent studies should increase the sample size to increase representativeness. Second, this was a cross-sectional study that was unable to explore the process by which individuals’ mental states are affected by DFDs. Therefore, longitudinal studies are needed. Third, deformities other than those in the sagittal plane may also cause depression in patients; for example, laterognathism of the mandible may also contribute to depression. To avoid the bias caused by this influence, this study did not include DFDs other than sagittal deformities. This may have resulted in the results of the study being limited to patients with Class II and Class III DFDs and may not be applicable to patients with other malformations. Therefore, future studies should also expand the scope of the discussion of types of DFDs. Furthermore, the present study did not discuss the effect of the severity of skeletal deformity on depression in patients, which is one of the limitations of this study. Future studies should explore the effect of the severity of skeletal deformity on patient depression based on quantitative indicators such as the ANB angle and wits value to deepen the understanding of the effect of DFD on patients’ psychological status. Finally, this study explored only the effects of demographic factors, disease type, and esthetic expectations on depression in patients with DFDs; however, numerous factors influence depression in patients with DFDs, and therefore, future studies should consider additional influencing factors.

## 5 Conclusions

This study explored the factors that may affect depression in DFD patients and revealed a greater risk of depression among patients who had a high school education, were diagnosed with Class III DFDs, and had higher esthetic expectations. When a patient’s deformity is more severe and their expectations for orthognathic surgery are greater, they may develop more severe depression. This study clarified the necessity of psychological interventions according to the type of disease and the level of patient expectations.

## Data Availability

The raw data supporting the conclusions of this article will be made available by the authors, without undue reservation.

## References

[1] Madhan S Nascimento GG Ingerslev J Cornelis M Pinholt EM Cattaneo PM. Health-related quality of life, jaw function and sleep-disordered breathing among patients with dentofacial deformity. J Oral Rehabil. (2024) 51:684–94. doi: 10.1111/joor.13619 38239176

[2] Rezaei F Masalehi H Golshah A Imani MM. Oral Health Related Quality of Life of Patients with Class Iii Skeletal Malocclusion before and after Orthognathic Surgery. BMC Oral Health. (2019) 19:289. doi: 10.1186/s12903-019-0989-9 31864336 PMC6925887

[3] Nascimento GG Goettems ML Cassiano LS Horta BL Demarco FF. Clinical and self-reported oral conditions and quality of life in the 1982 pelotas birth cohort. J Clin Periodontol. (2021) 48:1200–7. doi: 10.1111/jcpe.13512 34169558

[4] Wei HP Liu ZX Zang JJ Wang XD. Surgery-first/early-orthognathic approach may yield poorer postoperative stability than conventional orthodontics-first approach: A systematic review and meta-analysis. Oral Surg Oral Med Oral Pathol Oral Radiol. (2018) 126:107–16. doi: 10.1016/j.oooo.2018.02.018 29631986

[5] Maden A Akbulut N Balel Y. Comorbidity of body dysmorphic disorder and obsessive-compulsive disorder in orthognathic surgery patients. Eur J Ther. (2024) 30(5):606–15. doi: 10.58600/eurjther2254

[6] Nie JH Zhang Y Ma J Xue Q Hu M Qi HC. Major depressive disorder elevates the risk of dentofacial deformity: A bidirectional two-sample mendelian randomization study. Front Psychiatry. (2024) 15:1442679. doi: 10.3389/fpsyt.2024.1442679 39140105 PMC11319251

[7] Phillips C Bennett ME Broder HL. Dentofacial disharmony: psychological status of patients seeking treatment consultation. Angle Orthodontist. (1998) 68:547–55. 10.1043/0003-3219(1998)068<0547:DDPSOP>2.3.CO;2 PMC3612954 9851353

[8] Silva S Teixeira V Ferreira AP Ustrell-Torrent MJ. The reason of the psychological intervention in dentofacial deformity. Rev Portuguesa Estomatol Med Dentaria E Cirurgia Maxilofacial. (2016) 57:171–6. doi: 10.1016/j.rpemd.2016.03.003

[9] Sebastiani AM Gerber JT Bergamaschi IP Petinati MF Meger MN da Costa DJ. Individuals requiring orthognathic surgery have more depression and pain than controls. Braz Oral Res. (2021) 35:e091. doi: 10.1590/1807-3107bor-2021.vol35.0091 34378762

[10] Mota-Veloso I Ramos-Jorge J Freitas LRP Ferreira FO Ramos-Jorge ML Paiva SM. The prevalence of malocclusion is higher in schoolchildren with signs of hyperactivity. Am J Orthodontics Dentofacial Orthopedics. (2021) 159:653–9. doi: 10.1016/j.ajodo.2019.11.027 33658172

[11] da Motta TP Owens J Abreu LG Debossan SAT Vargas-Ferreira F Vettore MV. Malocclusion characteristics amongst individuals with autism spectrum disorder: A systematic review and meta-analysis. BMC Oral Health. (2022) 22:341. doi: 10.1186/s12903-022-02366-0 35948958 PMC9367144

[12] Kovalenko A Slabkovskaya A Drobysheva N Persin L Drobyshev A Maddalone M. The association between the psychological status and the severity of facial deformity in orthognathic patients. Angle Orthodontist. (2012) 82:396–402. doi: 10.2319/060211-363.1 22007634 PMC8865818

[13] Collins B Gonzalez D Gaudilliere DK Shrestha P Girod S. Body dysmorphic disorder and psychological distress in orthognathic surgery patients. J Oral Maxillofac Surg. (2014) 72:1553–8. doi: 10.1016/j.joms.2014.01.011 24582136

[14] Yang Y Ding RJ Hu D Zhang F Sheng L. Reliability and validity of a chinese version of the hads for screening depression and anxiety in psycho-cardiological outpatients. Compr Psychiatry. (2014) 55:215–20. doi: 10.1016/j.comppsych.2013.08.012 24199886

[15] Hagen KB Aas T Kvaloy JT Eriksen HR Soiland H Lind R. Fatigue, anxiety and depression overrule the role of oncological treatment in predicting self-reported health complaints in women with breast cancer compared to healthy controls. Breast. (2016) 28:100–6. doi: 10.1016/j.breast.2016.05.005 27262826

[16] Gore FM Bloem PJN Patton GC. Global burden of disease in young people aged 10-24 years: A systematic analysis (Vol 377, pg 2093, 2011). Lancet. (2011) 378:486–. doi: 10.1016/S0140-6736(11)60512-6 21652063

[17] Mendes VM Diluiso G Kamdem CJ Goulliart S Schettino M Dziubek M. Prevalence of psychiatric disorders in aesthetic surgery. Ann Plast Surg. (2023) 91:413–21. doi: 10.1097/sap.0000000000003682 37713148

[18] Kessler RC Ruscio AM Shear K Wittchen HU. Epidemiology of anxiety disorders. In: Stein MB Steckler T, editors. Behavioral Neurobiology of Anxiety and Its Treatment, vol. 2 (2010). p. 21–35. Current Topics in Behavioral Neurosciences. (Germany: Springer Berlin, Heidelberg). 21309104

[19] Torales J Barrios I González I. Oral and dental health issues in people with mental disorders. Medwave. (2017) 17:7045. doi: 10.5867/medwave.2017.08.7045 28937973

[20] Nascimento GG Gastal MT Leite FRM Quevedo LA Peres KG Peres MA. Is there an association between depression and periodontitis? A birth cohort study. J Clin Periodontol. (2019) 46:31–9. doi: 10.1111/jcpe.13039 30499588

[21] Kisely S Sawyer E Siskind D Lalloo R. The oral health of people with anxiety and depressive disorders a systematic review and meta-analysis. J Affect Disord. (2016) 200:119–32. doi: 10.1016/j.jad.2016.04.040 27130961

[22] Frejman MW Vargas IA Rösing CK Closs LQ. Dentofacial deformities are associated with lower degrees of self-esteem and higher impact on oral health-related quality of life: results from an observational study involving adults. J Oral Maxillofac Surg. (2013) 71:763–7. doi: 10.1016/j.joms.2012.08.011 22995696

[23] Barros A Mascarenhas P Botelho J MaChado V Balixa G Lopes LB. Autism spectrum disorders and malocclusions: systematic review and meta-analyses. J Clin Med. (2022) 11:2727. doi: 10.3390/jcm11102727 35628854 PMC9147636

[24] Basso IB Gonçalves FM Martins AA Schroder AGD Taveira KVM Stechman J. Psychosocial changes in patients submitted to orthodontic surgery treatment: A systematic review and meta-analysis. Clin Oral Investigations. (2022) 26:2237–51. doi: 10.1007/s00784-021-04304-w 34817686

[25] MaChado NC Gerber JT dos Santos KM Bergamaschi IP Meger MN da Costa DJ. Association of the estrogen receptor gene with oral health-related quality of life in patients with dentofacial deformities. Braz Oral Res. (2022) 36:e089. doi: 10.1590/1807-3107bor-2022.vol36.0089 35830136

[26] Häberle A Alkofahi H Qiao J Safer DL Mittermiller PA Menorca R. Body image disturbance and obsessive-compulsive disorder symptoms improve after orthognathic surgery. J Oral Maxillofac Surg. (2020) 78:2054–60. doi: 10.1016/j.joms.2020.07.021 32810443

[27] Nie P Teng F Wang F Shen GF Zhu M Yu DD. Correlation between esthetic expectations for orthognathic treatment and psychological characteristics among chinese adult patients. Am J Orthodontics Dentofacial Orthopedics. (2021) 160:94–100. doi: 10.1016/j.ajodo.2020.03.034 33906775

[28] Yu DD Wang F Wang XD Fang B Shen SG. Presurgical motivations, self-esteem, and oral health of orthognathic surgery patients. J Craniofacial Surg. (2013) 24:743–7. doi: 10.1097/SCS.0b013e318285d5a4 23714871

[29] Meade EA Inglehart MR. Young patients’ Treatment motivation and satisfaction with orthognathic surgery outcomes: the role of “Possible selves’’. Am J Orthodontics Dentofacial Orthopedics. (2010) 137:26–34. doi: 10.1016/j.ajodo.2008.03.022 20122427

[30] Möller J Mohr S Unterhorst K Stirn A. Plastic esthetic surgical operations to serve the psyche? Differences between plastic esthetic surgery patients with and without mental stress with respect to motives for surgery, personality, body perception, anxiety and previous history. J Fur Asthetische Chirurgie. (2017) 10:175–81. doi: 10.1007/s12631-017-0097-0

[31] Caplin J Han MD Miloro M Allareddy V Markiewicz MR. Interceptive dentofacial orthopedics (Growth modification). Oral Maxillofac Surg Clinics North America. (2020) 32:39. doi: 10.1016/j.coms.2019.08.006 31699583

[32] Brunault P Battini J Potard C Jonas C Zagala-Bouquillon B Chabut A. Orthognathic surgery improves quality of life and depression, but not anxiety, and patients with higher preoperative depression scores improve less. Int J Oral Maxillofac Surg. (2016) 45:26–34. doi: 10.1016/j.ijom.2015.07.020 26359548

[33] Ahorsu DK Adjaottor ES Yeboah FA Opoku Y. Mental health challenges in academia: comparison between students of the various educational levels in Ghana. J Ment Health. (2021) 30:292–9. doi: 10.1080/09638237.2020.1739253 32168994

[34] Chazelle E Lemogne C Morgan K Kelleher CC Chastang JF Niedhammer I. Explanations of educational differences in major depression and generalised anxiety disorder in the irish population. J Affect Disord. (2011) 134:304–14. doi: 10.1016/j.jad.2011.05.049 21676469

[35] Hakulinen C Musliner KL Agerbo E. Bipolar disorder and depression in early adulthood and long-term employment, income, and educational attainment: A nationwide cohort study of 2,390,127 individuals. Depression Anxiety. (2019) 36:1080–8. doi: 10.1002/da.22956 31508865

[36] Shuhua Zhang TC. A media perception survey of youth group's appearance anxiety and its reality influence study - an analysis based on the base model of personal information processing. Journalism Lovers (2022) 04:15–19. doi: 10.16017/j.cnki.xwahz.2022.04.027

[37] Burden DJ Hunt O Johnston CD Stevenson M O’Neill C Hepper P. Psychological status of patients referred for orthognathic correction of skeletal ii and iii discrepancies. Angle Orthodontist. (2010) 80:43–8. doi: 10.2319/022709-114.1 PMC8978754 19852638

[38] Sen E Duran H Sari M Akbulut N Demir O. Orthognathic surgery improves quality of life: A survey clinical study. BMC Oral Health. (2024) 24:844. doi: 10.1186/s12903-024-04638-3 39054469 PMC11270847

[39] Geramy A Nazarifar AM Shahroudi AS Sheikhzadeh S. Oral health-related quality of life following orthognathic surgery for class iii correction its relationship with cephalometric changes. Int J Oral Maxillofac Surg. (2019) 48:1434–9. doi: 10.1016/j.ijom.2019.03.899 31122800

[40] Borzabadi-Farahani A Eslamipour F Shahmoradi M. Functional needs of subjects with dentofacial deformities: A study using the index of orthognathic functional treatment need (Ioftn). J Plast Reconstructive Aesthetic Surg. (2016) 69:796–801. doi: 10.1016/j.bjps.2016.03.008 27068664

[41] Duarte V Zaror C Villanueva J Andreo M Dallaserra M Salazar J. Oral health-related quality of life changes in patients with dentofacial deformities class ii and iii after orthognathic surgery: A systematic review and meta-analysis. Int J Environ Res Public Health. (2022) 19:1940. doi: 10.3390/ijerph19041940 35206128 PMC8872566

[42] Pikoos TD Rossell SL Tzimas N Buzwell S. Assessing unrealistic expectations in clients undertaking minor cosmetic procedures: the development of the aesthetic procedure expectations scale. Facial Plast Surg Aesthetic Med. (2021) 23:263–9. doi: 10.1089/fpsam.2020.0247 32881596

[43] Honigman RJ Phillips KA Castle DJ. A review of psychosocial outcomes for patients seeking cosmetic surgery. Plast Reconstructive Surg. (2004) 113:1229–37. doi: 10.1097/01.Prs.0000110214.88868.Ca PMC1762095 15083026

[44] Sarcu D Adamson P. Psychology of the facelift patient. Facial Plast Surg. (2017) 33:252–9. doi: 10.1055/s-0037-1598071 28571061

[45] Jingyi Shi XW Wang L. Relationship between fear of negative evaluation and appearance anxiety among college students: the mediating role of dysfunctional attitude. China J Health Psychol. (2023) 31:1558–64. doi: 10.13342/j.cnki.cjhp.2023.12.026

[46] Beck AT Dozois DJA. Cognitive therapy: current status and future directions. In: Caskey CT, editor. Annual Review of Medicine, vol. 62 (2011) United States: Palo Alto, Calif., Annual Reviews, Inc. p. 397–409. 10.1146/annurev-med-052209-100032 20690827

