# Supplementary material for: Effects of individuals’ esthetic expectations and the classifications of dentofacial deformities on patients’ depression: a cross-sectional study
Source: Front Psychiatry. 2025 Mar 20;16:1505961. doi: 10.3389/fpsyt.2025.1505961 (PMC11967367; doi:10.3389/fpsyt.2025.1505961)
Supplement: Supplementary file 1 [file Table1.docx]

**Questionnaire**

**I. Basic information**

1. Your gender

🞐Men 🞐Women

1. Your age ( )
2. Your educational background

🞐Junior high school and below 🞐High school education 🞐College degree 🞐Bachelor degree 🞐Master degree and above

1. Your monthly income (Yuan)

🞐≤2000 🞐2001-5000 🞐5001-10000 🞐10001-15000 🞐≥15000

1. Your marriage status

🞐Married 🞐Unmarried 🞐Divorced or other

**II. measurement of esthetic expectations**

The current facial appearance and the expected facial appearance after surgery were presented as 2 statements, followed by a visual analog scale: (1) from 0 to 10, how do you rate your current facial appearance? (T1); (2) from 0 to 10, how do you rate the facial appearance you expect to achieve after the orthognathic surgery? (T2). The esthetic expectations were therefore expressed as the T2 − T1.

0 10

**III. Depression**

In the past two weeks, how often have the following symptoms been present in your life?

| **Questions** | **Not at All** | **Several Days** | **More Than Half the Days** | **Almost Every Day** |
| --- | --- | --- | --- | --- |
| 1. Little interest or pleasure in doing things. | 0 | 1 | 2 | 3 |
| 2. Feeling down, depressed or hopeless. | 0 | 1 | 2 | 3 |
| 3. Trouble falling asleep, staying asleep, or sleeping too much. | 0 | 1 | 2 | 3 |
| 4. Feeling tired or having little energy. | 0 | 1 | 2 | 3 |
| 5. Poor appetite or overeating. | 0 | 1 | 2 | 3 |
| 6. Feeling bad about yourself - or that you’re a failure or have let yourself or your family down. | 0 | 1 | 2 | 3 |
| 7. Trouble concentrating on things, such as reading the newspaper or watching television. | 0 | 1 | 2 | 3 |
| 8. Moving or speaking so slowly that other people could have noticed. Or, the opposite - being so fidgety or restless that you have been moving around a lot more than usual. | 0 | 1 | 2 | 3 |
| 9. Thoughts that you would be better off dead or of hurting yourself in some way. | 0 | 1 | 2 | 3 |
