# Supplementary material for: Effects of individuals’ esthetic expectations and the classifications of dentofacial deformities on patients’ depression: a cross-sectional study
Source: Front Psychiatry. 2025 Mar 20;16:1505961. doi: 10.3389/fpsyt.2025.1505961 (PMC11967367; doi:10.3389/fpsyt.2025.1505961)
Supplement: Supplementary file 2 [file Table2.docx]

**Table 1multivariate regression analysis of esthetic expectations**

| Esthetic expectations | | B | Se | Wals | P | OR | 95%CI | |
| --- | --- | --- | --- | --- | --- | --- | --- | --- |
|  | |  |  |  |  |  | Lower | Upper |
| Low | Constant | 0.47 | 0.18 | 6.60 | 0.01* |  |  |  |
|  | Class III DFD (REF: Class II DFD) | 1.44 | 0.57 | 6.44 | 0.01* | 4.21 | 1.39 | 12.77 |
| High | Constant | 0.15 | 0.20 | 0.61 | 0.43 |  |  |  |
|  | Class III DFD (REF: Class II DFD) | 1.40 | 0.58 | 5.78 | 0.02* | 4.07 | 1.30 | 12.80 |
| Extremely high | Constant | 1.28 | 0.16 | 61.13 | <0.001** |  |  |  |
|  | Class III DFD (REF: Class II DFD) | 1.56 | 0.54 | 8.32 | <0.001** | 4.74 | 1.65 | 13.67 |

Note: *indicated P<0.05, **indicated P<0.01
